# SMAD7 rs4939827 variant contributes to colorectal cancer risk in Chinese population

**DOI:** 10.18632/oncotarget.17065

**Published:** 2017-04-12

**Authors:** Chunze Zhang, Xichuan Li, Wenzheng Fu, Yijia Wang, Tao Wang, Wenhong Wang, Shuo Chen, Hai Qin, Xipeng Zhang

**Affiliations:** ^1^ Department of Colorectal Surgery, Tianjin Union Medical Center, Tianjin 300121, China; ^2^ Department of Immunology, Biochemistry and Molecular Biology, 2011 Collaborative Innovation Center of Tianjin for Medical Epigenetics, Tianjin Key Laboratory of Medical Epigenetics, Tianjin Medical University, Tianjin 300070, China; ^3^ Department of Pathology, Tianjin Union Medical Center, Tianjin 300121, China; ^4^ Department of Imaging, Tianjin Union Medical Center, Tianjin 300121, China

**Keywords:** colorectal cancer, SMAD7, rs4939827, meta-analysis

## Abstract

A genome-wide association study identified a common genetic variant rs4939827 at 18q21 in SMAD7 to be related with colorectal cancer (CRC) risk with OR=1.2 and *P* =7.80E-28. Until recently, several meta-analysis studies have been conducted, and reported significant association between rs4939827 and CRC risk. However none of these studies evaluated the potential association between rs4939827 and CRC risk in Chinese population. In this study, we evaluated this association by a meta-analysis using 12077 samples including 5816 CRC cases and 6261 controls. In the end, we identified the T allele of rs4939827 to be significantly related with an increase CRC risk (*P*=2.22E-05, OR=1.14, 95% CI 1.07-1.21) in Chinese population.

## INTRODUCTION

It is reported that colorectal cancer (CRC) is the third most common type of cancer and the leading cause of cancer death, which could cause about 600,000 deaths in the world annually [1–2]. Evidence shows that CRC is a common human complex disease, which is considered to be caused by the interactions between genetic variants and environmental factors [1–2]. It is reported that some factors including allele frequencies, specific linkage disequilibrium structure, and special genetic and environmental backgrounds may cause the risk alleles variation to CRC risk in different populations [3]. Meanwhile, evidence showed racial differences in the incidence of colorectal cancer [4–6]. The incidence in native Chinese is significantly lower than in Chinese-Americans, who have similar rates with the native Americans [5]. Virk et al. reported the significant differences in the incidence of colorectal cancers in various racial subgroups in British Columbia [5]. The incidence in Chinese Canadians population is significantly lower than in Caucasian Canadians and South Asian Canadians [5]. There are also different survival and clinicopathologic features in colorectal cancer in African American, Caucasian, and Chinese patients [7]. All these findings indicate that it is still necessary to evaluate a specific variant in a specific population, which would be informative to reveal the disease mechanism [3].

Since 2007, several large-scale genome-wide association studies (GWAS) have been performed to detect common CRC genetic variants [8–14]. In these above studies, Tenesa et al. identified a common genetic variant rs4939827 at 18q21 in SMAD7 to be associated with CRC risk with OR=1.2 and *P* =7.80E-28 [9].

To further verify the original finding from above study, some genetic association studies have investigated the association between rs4939827 and CRC risk in other populations. However these studies reported both positive and negative association results. Until recently, several meta-analysis studies have also been conducted, and reported significant association between rs4939827 and CRC risk [15–17]. However none of these studies evaluated the potential association between rs4939827 and CRC risk in Chinese population. In this study, we aim to evaluate this association in Chinese population.

## RESULTS

### Study characteristics

We selected 4 independent case-control association studies from previous meta-analyses [15–17]. Song et al. selected 12 publications with 25 case-control studies including 19 studies in European population, 1 study in the mixed population, and 5 studies in Asian population, which included 4 studies in Chinese population [15]. Yao et al. selected 21 case-control studies including 14 studies in European population, 1 study in African population, and 6 studies in Asian population, which included 3 studies in Chinese population [16]. Hong et al. selected 12 case-control studies, which only included 2 studies in Asian population and Chinese population [17]. We also selected another two independent case-control association studies using Google Scholar and Baidu Scholar databases. All these 6 studies evaluated the potential association between rs4939827 and CRC risk in Chinese population using a total of 12077 samples including 5816 CRC cases and 6261 controls. We described the main characteristics of these 6 studies in Table 1.

**Table 1:** Main characteristics of 6 studies

Study	Year	Allele	MAF	OR [95% CI]	Population	Ethnicity	Cases	Controls	Family history	Mean age	HWE	*P* value
Xiong [19]	2010	T vs. C	0.17	1.17 [1.05-1.31]	Han Chinese	Han	2124	2124 (P)	NA	56.9±11.8 in cases56.4±11.3 in controls	Yes	5.70E-03
Ho [20]	2011	T vs. C	0.35	1.18 [1.01-1.38]	Hong Kong Chinese	NA	892	890 (H)	No	66.75±12.25 in cases in phase 166.43±12.21 in cases in phase 2	Yes	0.037
Li [21]	2011	T vs. C	0.26	0.90 [0.63-1.28]	Han Chinese	Han	138	168 (NA)	NA	52.53±13.405 in cases59.26±15.854 in controls	Yes	0.771
Song [15]	2012	T vs. C	0.20	1.26 [1.05-1.51]	Han Chinese	Han	641	1037 (H)	NA	56.31±12.59 in cases57.24±10.86 in controls	Yes	0.01
Thean [22]	2012	T vs. C	NA	1.13 [0.99-1.28]	Singapore Chinese	NA	991	993 (P)	No	70 in cases69 in controls	Yes	0.068
Tan [23]	2016	T vs. C	0.28	1.05 [0.92-1.20]	Han Chinese	Han	1030	1049 (P)	No	60.9±11.0 in cases59.7±8.2 in controls	Yes	0.94

MAF, minor allele frequency for T allele in controls; NA, not reported; OR, odds ratio; CI, confidence interval; HWE, Hardy Weinberg equilibrium; H or P, controls are hospital (H) or population (P) based.

### Heterogeneity test results

The heterogeneity test shows that tau^2 = 0, H = 1 [1; 1.94], I^2 = 0% [0%; 73.3%], Cochran's Q statistic = 4.76, degrees of freedom = 5, and *P*=0.4462. All these results show no statistically significant heterogeneity.

### Meta-analysis results

**Figure 1:**
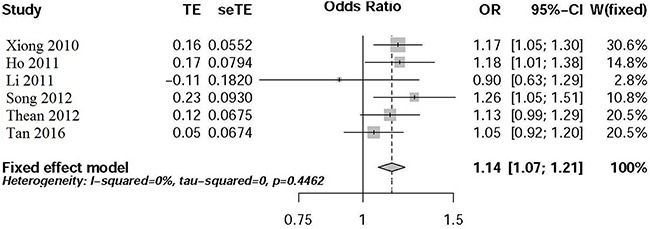
Forest plot for meta-analysis of rs7014346 variant in Chinese population

We applied the fixed-effect model to perform the meta-analysis. As described in Figure 1, the T allele of rs4939827 was significantly related with an increase CRC risk (*P*=2.22E-05, OR=1.14, 95% CI 1.07-1.21) in Chinese population.

### Publication bias

**Figure 2:**
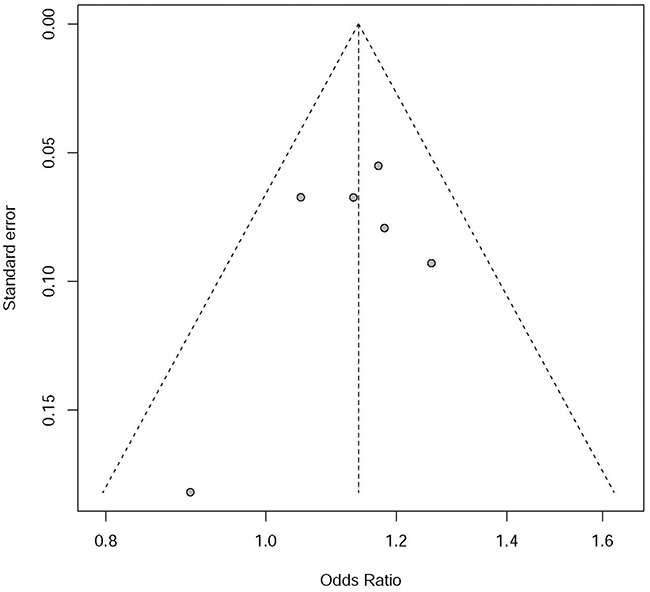
Funnel plot for publication bias analysis of rs7014346 in Chinese population

As described in Figure 2, no significant asymmetry was observed in the funnel plot. Linear regression test of funnel plot asymmetry suggested that there was no significant publication bias in our meta-analysis *P*=0.496.

### Subgroup analysis

In Table 1, we selected six studies including four studies in Han Chinese population, one in Hong Kong Chinese, and one in Singapore Chinese. In 2009, evidence shows substantial genetic variation among Han Chinese population [18]. It would be helpful to consider geographical area. Here, we further performed a subgroup analysis including the Han Chinese subgroup, as well as combined Hong Kong and Singapore Chinese subgroup.

In Han Chinese subgroup, the heterogeneity test shows that tau^2 = 0.0034; H = 1.23 [1; 2.07]; I^2 = 33.6% [0%; 76.6%], Cochran's Q statistic = 4.52, degrees of freedom = 3, and *P*=0.2109. All these results show no statistically significant heterogeneity. We applied the fixed-effect model to perform the meta-analysis. We identified that the T allele of rs4939827 was significantly related with an increase CRC risk (*P*=1.10E-03, OR=1.13, 95% CI 1.05-1.22) in Han Chinese subgroup.

In combined Hong Kong and Singapore Chinese subgroup, the heterogeneity test shows that tau^2 = 0; H = 1; I^2 = 0%, Cochran's Q statistic = 0.17, degrees of freedom = 1, and *P*=0.6777. All these results show no statistically significant heterogeneity. We again applied the fixed-effect model to perform the meta-analysis. We identified that the T allele of rs4939827 was significantly related with an increase CRC risk (*P*=6.30E-03, OR=1.15, 95% CI 1.04-1.27) in combined Hong Kong and Singapore Chinese subgroup.

## DISCUSSION

Tenesa et al. identified rs4939827 to be associated with CRC risk with OR=1.2 and *P* =7.80E-28 [9]. However following studies reported inconsistent results. Until recently, several meta-analysis studies have been conducted, and reported significant association between rs4939827 and CRC risk [15–17]. In their meta-analysis, Song et performed a stratified analysis. They observed significant heterogeneity in European and Asian populations [15]. In our study, we did not observed significant heterogeneity in Chinese population. In their meta-analysis, Yao et al. selected 21 case-control studies including 14 studies in European population, 1 study in African population, and 6 studies in Asian population, which included 3 studies in Chinese population [16]. A subgroup analysis indicated significant correlation between rs4939827 and CRC risk in the Caucasian group [16]. However, this correlation was not observed in the Asian and African groups [16]. In their meta-analysis, Hong et al. selected 12 case-control studies, which only included 2 studies in Asian population and Chinese population [17]. Their stratified analyses also show that the heterogeneity in the European population is higher than that in the Asian population [17].

Until recently, at least 6 independent case-control association studies have been conducted to investigate the association between rs4939827 and CRC risk in Chinese population. Three studies reported positive association results [15, 19–20], and another three studies reported negative association results [21–23]. Until recently, three meta-analysis studies have been conducted [15–17]. However none of these studies evaluated the potential association between rs4939827 and CRC risk in Chinese subgroup analysis [15–17]. In this study, we evaluated this association by a meta-analysis, and identified significant association between rs4939827 and CRC in Chinese population.

In 2009, Chen et al. analyzed 350000 SNPs in over 6000 Han Chinese samples [18]. They identified substantial genetic variation among Han Chinese population [18]. Based on this consideration, we further performed a subgroup analysis in the Han Chinese subgroup, and the combined Hong Kong and Singapore Chinese subgroup. Our results are consistent with previous findings. The heterogeneity in Han Chinese subgroup (I^2 = 33.6%) is higher compared with that in combined Hong Kong and Singapore Chinese subgroup (I^2 = 0%). Interestingly, the T allele of rs4939827 was significantly related with an increase CRC risk in both Han Chinese subgroup, and the combined Hong Kong and Singapore Chinese subgroup.

Evidence shows decreased SMAD7 expression in CRC cases [24]. Evidence also supports a role for SMAD7 in sustaining colon tumorigenesis [25]. SMAD7 could block TGFβ signaling [26]. Boulay et al. previously evaluated the clinical relevance of the deletion of SMAD7, and identified SMAD7 to be a prognostic marker in CRC patients and play an important role in tumor suppression [26]. The loss of SMAD7 could cause carcinoma cells more sensitive [26]. The gain of SMAD7 could cause TGFβ resistance [26]. Phipps et al. investigated the association between rs4939827 and survival of 2611 individuals with CRC [27]. The results indicated that the minor allele in rs4939827 was significantly associated with reduced overall survival and disease-specific survival. All these findings show that rs4939827 variant could affect CRC progression [27]. In addition to the rs4939827 variant, evidence also showed that both rs12953717 and rs11874392 variants were also associated with risk of CRC [24].

## MATERIALS AND METHODS

### Search strategy

In the PubMed database, we selected the potential studies using the key words ‘SMAD7’ + ‘colorectal cancer’ + ‘meta’ (n=9), and ‘rs4939827’ + ‘colorectal cancer’ + ‘meta’ (n=6). We also applied Google Scholar (https://scholar.google.com/), Chinese National Knowledge Infrastructure (CNKI), Baidu Scholar (http://xueshu.baidu.com/) to search manually all associated publications citing the original CRC GWAS [9]. Here, we limit our analysis in Chinese populations including a native or inhabitant of China or a person of Chinese ancestry. There is no limitation on language searched. We included the colorectal cancer cases and excluded the colorectal adenomatous polyps cases.

### Data extraction

The selected studies should provide (1) a case-control design in Chinese population; (2) association between rs4939827 and CRC risk; (3) odds ratio (OR) with 95% confidence interval (CI) for allele model T vs. C; or (4) sufficient data to calculate the OR and 95% CI for allele model The following items were extracted (1) the name of the first author; (2) the year of publication; (3) the population and ethnicity; (4) the numbers of CRC cases and controls; (5) the OR with 95% CI; All the possible studies are excluded: abstracts and reviews; and duplicated publications. More detailed information is described in previous studies [15–17, 28–30].

### Hardy-Weinberg equilibrium test

In general, the genetic case-control association studies may be spurious if the distribution of genotypes in the healthy control groups deviates from Hardy-Weinberg equilibrium (HWE). A variant strongly associated with the disease would sometimes show deviance from HWE in case samples [31]. Thus all the selected studies evaluated the potential deviates from HWE in control groups [31]. If the original study provides the control genotype number, we calculated the HWE by a chi-square test [32–33]. If the original study does not provide the control genotype number, we extracted the HWE information from the original studies.

### Statistical analysis

The potential heterogeneity is evaluated by Cochran's Q test [15–17, 28–30, 34–41]. If there is no significant heterogeneity, the pooled OR is calculated by a fixed effect model; otherwise by a random-effect model [15–17, 28–30, 35–41]. Z test is used to determine the significance of pooled OR [28–30]. A funnel plot is used to investigate potential publication bias as described in previous studies [15–17, 28–30, 35–41]. A linear regression based approach is used to test for publication bias, and provide statistical evidence [28–30, 35–41]. All statistical analyses were performed using R, and the significance level is 0.05.
